# Voluntary Hospitals Commission

**Published:** 1923-04

**Authors:** 


### VOLUNTARY HOSPITALS COMMISSION.

### AN ENCOURAGING REPORT.

JUST too late for our last issue, the Voluntary Hospitals Commission presented an Interim Report (Stationery Office, 9d.) on the administration of the Government grant of £500,000 up to December 31, 1922. On the basis of the ratio of aggregate deficits, the Commission have allocated £225,000 to the Metropolitan hospitals and £275,000 to the hospitals in the rest of England, Wales and Scotland. The total sum distributed, up to December 31, 1922, is £373,013.

### The Pound for Pound Stipulation.

The grants have prevented the closing of further beds, and in many cases have enabled hospitals to reopen closed beds. So far, they have been made in respect of the deficits on maintenance accounts for the year 1921. Any balance that may be available when these claims have been disposed of will be used as far as possible to assist hospitals which have heavy accumulated deficits. The Treasury condition that the disbursement of the grant should proceed *pro rata* against fresh money raised from voluntary sources has proved a valuable incentive to the hospitals to make special efforts, and they have raised in the aggregate a sum far larger than the grant administered by the Commission. In London, for example, the Combined Appeal organised by King Edward's Hospital Fund raised over £400,000.

### Local Committees.

In accordance with the recommendations of Lord Cave's Committee, the Commission have established Local Voluntary Hospital Committees in practically every part of England, Wales and Scotland. These Committees comprise representatives of the local authorities, the hospitals, the medical profession, and others directly nominated by the Commission. They have acted as local advisers to the Commission with regard to the applications for grants; they have also in some cases taken the initiative in raising new money from voluntary sources. Many have furnished valuable reports on the hospital position in their area. With the exception of London, where the King's Fund acted as the local committee, the county was taken as the basis of the local organisation :—

An exception to the general rule of the county basis was made in the case of a limited number of the larger county boroughs, and separate committees were accordingly established in Birmingham, Bristol, Liverpool, Manchester, Portsmouth, Plymouth, Salford and Sheffield. A few of the less densely populated counties with only a small number of hospitals were grouped and joint committees were formed for Berkshire, Buckinghamshire and Oxfordshire, Cumberland and Westmorland, East and West Ridings of Yorkshire, Northumberland, Newcastle and Gateshead, Leicestershire and Rutland. In a few English counties (mainly agricultural) in which the hospitals were not in need of financial assistance no Local Committees have yet been formed.

### The London Hospitals.

One of the first steps taken by the Commission was to ascertain the position of the Metropolitan hospitals :—

The King's Fund obtained in October, 1921, an estimate from all the London hospitals of the anticipated surplus or deficit on the year, and these figures indicated a probable gross aggregate deficit on the maintenance account for 1921 of £360,000, a formidable figure but substantially less than the aggregate deficit of £463,000 in 1920, as ascertained by Lord Cave's Committee. We came to the conclusion that the 1921 deficits should be treated as a first charge on the funds at our disposal, leaving the accumulated deficits from previous years to be dealt with later out of any balance which might be available when the 1921 claims had been met; and we accordingly decided to appropriate £90,000, or half the maximum amount available on this basis for London, to be applied in emergency grants to those hospitals which had exhausted their realisable assets and had been, or were likely to be, compelled to close beds.

### Free Legacies and Maintenance Income.

The Commission invariably followed the rule adopted by Lord Cave's Committee of reckoning "free" legacies (legacies, that is, not subject to trusts, or earmarked by the testator for special purposes) as part of the maintenance income :—

As it is the custom of many hospitals to carry all free lagacies to capital account, it followed that in many cases they did not receive 50 per cent. of the deficits as shown in their published accounts. But while it is a prudent policy in times of prosperity to invest free legacies, it is, in our view, unsound finance to increase investments when at the same time the hospital is paying on a growing bank overdraft a rate of interest higher than the yield of the investments; nor is it fair to the public to exaggerate the deficit on the maintenance account by carrying direct to endowment receipts which only differ from other donations in being paid posthumously. In London, thanks to the efforts of the Central Funds, hospital accounts are kept on a uniform basis designed to disclose the true financial position; but this practice has not been adopted by all the provincial hospitals, and in many cases the exclusion of free legacies from the maintenance income produces a misleading impression.

### Improvement "Very Marked."

On the whole there was found to be an improvement in the finances of the hospitals :—

Naturally, there were considerable variations between different areas, and in a few cases where adverse industrial conditions were specially acute the 1920 deficits were exceeded. But in general, the improvement was very marked, and there was good ground for expecting that the complete return would show much the same reduction of deficits in comparison with Lord Cave's estimate, as had been realised in London. On the whole, hospital incomes had been well maintained, and in a few areas, notably in Devonshire, appreciably increased; but the improvement was due in the main to the reduction of expenditure following the rapid fall in the cost of living.

### An Obligatory System of Accounts.

In his report Lord Cave emphasised the value of a uniform system of accounts, and the adoption of a standard form approved by the Commission has been made one of the conditions of earning the Government grant :—

The Commission recognised that the uniform system as as laid down by the Central Funds was too elaborate for use by most cottage hospitals; and a small committee was appointed to draft a simple scheme of account keeping which, while preserving the essential features of the uniform system, should yet be easily followed by those who are not trained accountants. It was found that the King's Fund had also appointed a committee to go into the question. A meeting was arranged between the two committees and a form was settled on the lines suggested by the King's Fund.

### Vitality of the Voluntary System.

Special reference is made to the steps taken in many areas to organise contributory schemes as a method of securing a new and permanent source of income for the hospitals. The Commission are engaged in collecting particulars of these various schemes, with a view to placing the information at the disposal of the various Local Committees. Reviewing the position of the voluntary hospitals generally, the Commission report :—" The results of our investigations and experience during the period under review show conclusively that the voluntary system possesses strong vitality, and that its chances of permanent survival are increasingly hopeful." The Commission observe as follows :—

" It i- striking proof of the vitality of the voluntary system that in a period of unprecedented unemployment and heavy taxation the total income of the hospitals has been so well maintained. There is every reason to hope that as soon as trade improves the great majority of the hospitals will be able to balance their budgets. The outlook, therefore, is hopeful, and indeed the recovery has been far more rapid than seemed likely when Lord Cave's Committee issued their report. But much still remains to be done and the voluntary system cannot yet be said to be completely out of danger.

### Need for Capital Expenditure.

Though the margin between income and expenditure is narrowing, it is still dangerously wide, and the difficulties of the post-war period have led to the indefinite postponement of much-needed capital expenditure :—

It should be borne in mind that no increase of hospital accommodation has been undertaken on any considerable scale since 1914, except perhaps the provision of increased accommodation for the nursing staffs, which have been expanded as a result of the recent shortening of the hours of work. There is no indication of any reduction in the long waiting lists to which Lord Cave's Committee called attention. These might to some extent be reduced by a more systematic distribution of patients ; but it cannot be maintained that the total accommodation is adequate to meet the increasing demands upon it. The provision of additional accommodation is essential if the success of the voluntary system in the future is to be secured.

### Fear of Government Interference.

In discussing the future of the Commission and the Local Voluntary Hospital Committees, the Commission point out that the association of the Commission with a Government Department and of the Local Committees with representatives of the local authorities had given rise to the apprehension that governmental or municipal control might eventually supervene, and that the voluntary system was thereby jeopardised. Events, however, have proved that the conditions governing the administration of the grant have proved a valuable stimulus to voluntary effort, and that the present organisation has done much to insure the maintenance of the voluntary system.

### The Future.

As regards the future the report points out that the continuance of the Local Committees will depend on the extent to which they justify themselves by their utility and command the confidence of the hospitals. So long as any considerable number of Local Committees continue to function as active organisations it seems desirable that some central body, such as the Commission, should continue in order to facilitate their co-operation ; and the Commission recommend that that body should retain its association with the Ministry of Health at any rate for the present.

